# Functional validation of a non-canonical HNF1B splice-site variant in MODY5

**DOI:** 10.3389/fendo.2026.1777094

**Published:** 2026-04-10

**Authors:** Xinran Gong, Li Zhang, Qiaobo Ma, Yingyu Zhang, Keyan Hu, Hongwei Jiang, Huifang Peng

**Affiliations:** 1 Henan Key Laboratory of Rare Diseases, Department of Endocrinology, The First Affiliated Hospital, and College of Clinical Medicine of Henan University of Science and Technology, Luoyang, China; 2 Department of Endocrinology and Metabolism, Anyang Steel General Hospital, Anyang, China

**Keywords:** HNF1B, minigene experiments, MODY5, renal cysts, WGS

## Abstract

**Background:**

Mutations in the *HNF1B* gene can cause developmental and functional abnormalities in multiple organs such as the pancreas, kidneys, and liver. The patient is a 19-year-old male with normal growth and development, presenting with a 9-year history of diabetes that remains poorly controlled despite intensive subcutaneous insulin therapy. His clinical features include surgically managed bilateral cataracts, hyperuricemia, and bilateral renal cysts. Family history is significant for diabetes in paternal and maternal uncles.

**Methods:**

This combination of early onset, multiorgan involvement, and familial pattern strongly suggests monogenic diabetes, indicating the need for genetic analysis to confirm the diagnosis and guide management. This study performed genetic sequencing on a patient suspected of maturity-onset diabetes of the young (MODY), followed by a Minigene assay on the identified variant of uncertain significance.

**Results:**

Trio-whole-genome sequencing (Trio-WGS) identified the *de novo HNF1B* splice-site variant (NM_000458.4: c.544+3_544+6delAAGT). The Minigene experiments indicate that this point mutation affects the splicing function of *HNF1B* gene with producing a truncated protein of 170 amino acids.

**Conclusion:**

In this study, we identified a *de novo* splicing mutation in the *HNF1B* gene through combined genomic analysis and functional verification, confirming that it leads to aberrant mRNA splicing. For patients with early-onset diabetes and extra-renal manifestations, if genetic screening for *HNF1B* exons and canonical splice sites yields negative results, the pathogenic potential of variants in introns and non-canonical splice sites should be carefully considered.

## 1 Introduction

MODY is a type of diabetes that typically presents in adolescents and young adults, usually before the age of 25, and is frequently associated with a positive family history. Historically, Maturity-Onset Diabetes of the Young (MODY) has been classified into 14 subtypes based on genetic mutations and clinical presentations (1). However, a recent study in the UK re-evaluating the pathogenicity of MODY genes indicated that *BLK*, *PAX4*, and *KLF11* should no longer be considered pathogenic causes of MODY, given the high frequency of their loss-of-function (LOF) variants in the general population (2). *HNF1B* functions as a transcription factor that regulates embryonic development by modulating the Wnt signaling pathway and interacting with other genes. Mutations in *HNF1B* lead to MODY5. Patients with this subtype frequently present with multiple anomalies of the genitourinary system, as well as hepatic dysfunction and electrolyte disturbances. Notably, approximately 50% of *HNF1B* mutations arise *de novo* (3). We report a case of a 19-year-old diabetic patient presenting with renal cysts and cataracts, in whom genetic testing identified a *de novo HNF1B* mutation. Furthermore, we conducted *in vitro* functional studies to further elucidate the pathogenic mechanisms of monogenic diabetes.

## 2 Materials and methods

### 2.1 Clinical ethics statement

The First Affiliated Hospital of Henan University of Science and Technology has obtained ethical approval for this experiment. And the written consent of the patient and his/her family had been obtained.

### 2.2 Clinical data

The present study collected the following clinical data: (1) general information, including sex, age, height, body weight, body mass index (BMI), and medical history, (2) laboratory investigations: biochemical profiles (liver function, renal function, urinary microalbumin, fasting blood glucose, C-peptide, uric acid, and routine blood tests), (3) imaging studies (ultrasonography) and family history investigation.

### 2.3 The trio-WGS sequencing

Peripheral blood samples (2 mL) were collected from the proband and parents, and Trio-WGS was performed using the DNBSEQ-T7 platform (BGI, China). Suspected variants were subsequently validated by Sanger sequencing. Sequencing data were aligned to the human reference genome (hg19/GRCh38). To exclude common single nucleotide polymorphisms (SNPs), variant frequencies were determined using data from the 1000 Genomes Project, the NHLBI Exome Sequencing Project (ESP), and the Exome Aggregation Consortium (ExAC). We utilized SIFT, PolyPhen-2, and MetaSVM to predict the pathogenicity of *HNF1B* variants. The reference sequence for *HNF1B* (NM_000458.4) was retrieved from GenBank. Variant interpretation was performed in accordance with the standards and guidelines established by the American College of Medical Genetics and Genomics and the Association for Molecular Pathology (ACMG/AMP).

### 2.4 Minigene plasmid construction and splicing assay

To investigate the effect of the *HNF1B* c.544 + 3_544 + 6delAAGT variant on pre-mRNA splicing, we constructed wild-type (WT) and mutant (MUT) minigene vectors. Two pairs of nested primers were designed for the minigene assays, with sequences as follows: 5’-tcaaagcatctgttcaataagca-3’ vs. 5’-aagcgttgttgggtctttgg-3’, and 5’-ggctaagacatcctccttgc-3’ vs. 5’-gccagaggatagtgtgtggg-3’. Using genomic DNA from a healthy individual as a template, a fragment containing *HNF1B* partial Intron 1 (553 bp), Exon 2 (200 bp), and partial Intron 2 (550 bp) was amplified via nested PCR and cloned into the pcMINI vector (containing a universal Exon A - Intron A - MCS - Intron B - Exon B cassette). Simultaneously, a fragment comprising *HNF1B* Exon 1 (344 bp), partial Intron 1 (757 bp), Exon 2 (200 bp), and partial Intron 2 (573 bp) was amplified and cloned into the pcMINI-N vector (containing a universal MCS - Intron B - Exon B cassette). Following restriction enzyme digestion, purification, ligation, transformation, and colony PCR screening, the recombinant vectors were validated by DNA sequencing. The validated vectors were then transiently transfected into HeLa and 293T cell lines using a liposome transfection reagent according to the manufacturer’s protocol. Cells were harvested 48 hours post-transfection. Total RNA was extracted using a commercial kit, and equal amounts of quantified RNA were reverse-transcribed into cDNA. PCR amplification was performed using vector-specific flanking primers: 5’-CTAGAGAACCCACTGCTTAC-3’ and 5’-GCCCTCTAGActggtcattccggct-3’ for the pcMINI vector; and 5’-CTAGAGAACCCACTGCTTAC-3’ and 5’-GCCCTCTAGActggtcattccggctc-3’ for the pcMINI-N vector. The PCR products were analyzed by agarose gel electrophoresis. Specific bands were excised, purified, and subjected to Sanger sequencing.

### 2.5 Literature search

To identify prior reports on the pathogenicity of this variant, we searched the ClinVar and PubMed databases using keywords including *HNF1B* and MODY5, especially focus on the c.544+3_544+6delAAGT mutation on *HNF1B* gene. And had considered the differences observed in different reference genome versions, as GRCh37 or GRCh38.

## 3 Results

### 3.1 Clinical characteristics of the cases

The patient was diagnosed with diabetes over nine years ago during a preoperative evaluation for circumcision, which revealed elevated blood glucose accompanied by polydipsia, dry mouth, and polyuria, but without polyphagia or weight loss. Laboratory tests at that time showed a Glycated Hemoglobin (HbA1c) level of 11.60% (4.50-6.30%) and a fasting plasma glucose of 17.33 mmol/L (3.90-6.00 mmol/L). Initial intensive insulin therapy was effective, with blood glucose remaining normal after insulin discontinuation. However, the patient subsequently refused regular blood glucose monitoring, and the long-term efficacy of glycemic control remains unknown. Four years ago, the patient was diagnosed with cataracts due to visual deterioration. At that time, blood glucose was 27.1 mmol/L and HbA1c was 18.50%, leading to the initiation of intensive insulin therapy with recombinant human insulin combined with insulin detemir, yet glycemic control remained suboptimal. Three years ago, the patient developed increased foamy urine, without symptoms of diabetic neuropathy such as limb numbness or intermittent claudication. One month ago, an outpatient examination revealed a serum uric acid level of 633.60 µmol/L (208.00-428.00 µmol/L).

Physical examination findings are as follows: height 180 cm, weight 55 kg, Body Mass Index (BMI) 16.98 kg/m^2^, and heart rate 79 beats/min. The patient was born full-term via vaginal delivery, and his growth and development have been comparable to those of his peers. He exhibits normal penile development, with a foreskin that is retractable. The urethral meatus shows no redness, swelling, or discharge. No abnormalities were detected in the bilateral testes or epididymides, the cremasteric reflex is normal, and no other positive findings were observed.

Laboratory results are as follows: HbA1c 8.60%, fasting plasma glucose 9.69 mmol/L, fasting C-peptide 0.273 ng/mL (0.200-4.000 ng/L), serum creatinine 110.80 µmol/L (44.20-94.60 µmol/L), urinary microalbumin 117.30 mg/L (0-20.00 mg/L), urea 9.23 mmol/L (3.80-6.10 mmol/L), red blood cell count 5.75×10^12^/L (4.30×10^12^/L-5.80×10^12^/L) and uric acid 633.60 µmol/L. Autoantibody testing revealed positive results for insulin autoantibodies (IAA), and negative results for glutamic acid decarboxylase (GAD), insulinoma-associated antigen-2 (IA-2), and zinc transporter 8 (ZnT8) autoantibodies. Renal ultrasound revealed diffusely altered renal echogenicity and a left renal cyst (0.6 cm×0.4 cm).

Past medical history and family history are as follows: The patient underwent cataract surgery four years ago. There is a family history of type 2 diabetes (T2DM) in the maternal grandfather and a paternal uncle, though the exact time of onset is unknown. They are currently managing their blood glucose with oral antihyperglycemic agents, including metformin, empagliflozin, and acarbose. The patient’s renal development is normal, but routine renal function monitoring had not been performed previously.

### 3.2 The trio-WGS results of the patient

**Figure 1:**
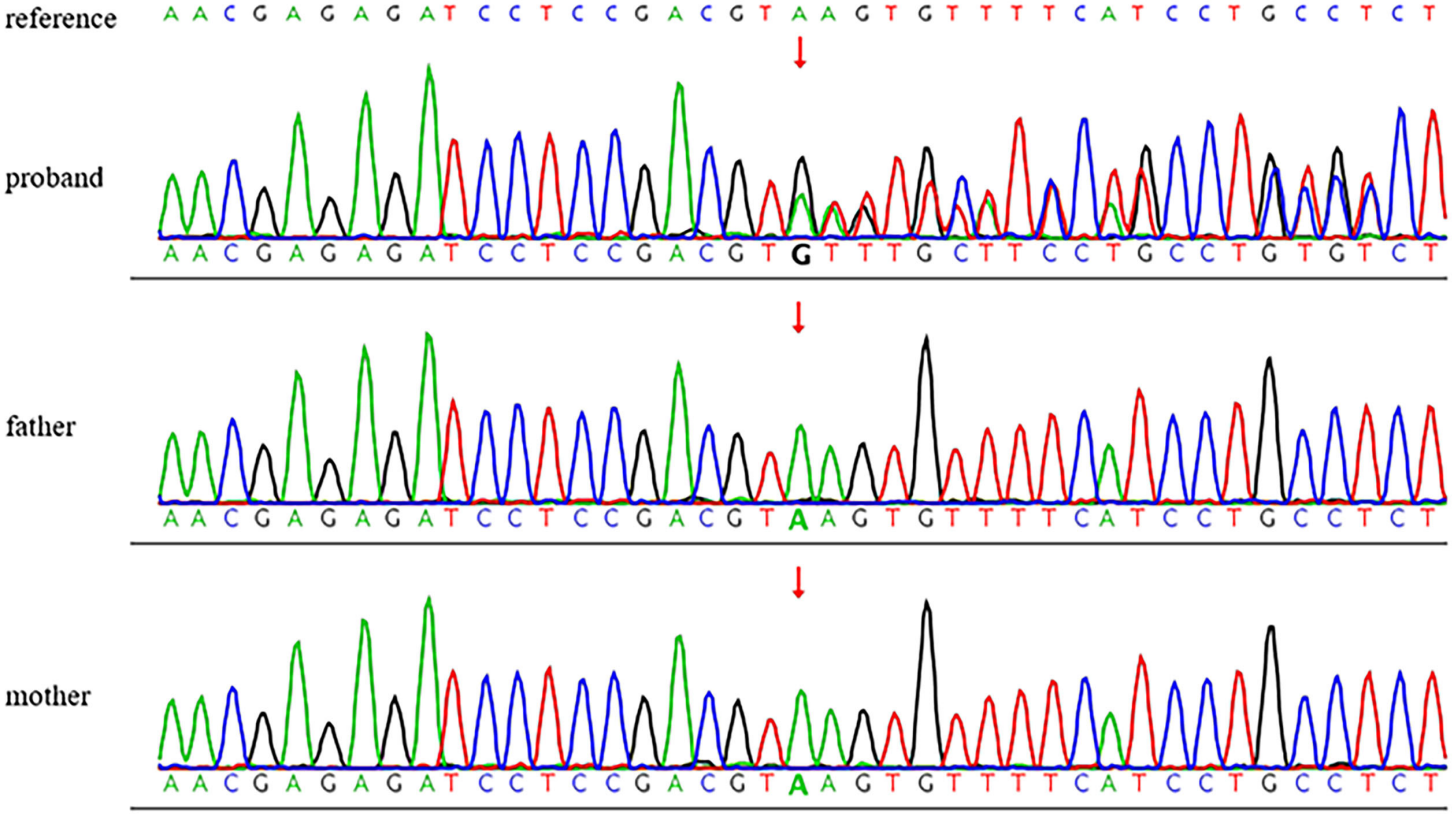
Information of the Sanger sequencing results: The reference sequence of *HNF1B* was derived from GenBank and the version number was NM_000458.4. Proband: Discovery of *HNF1B* gene c.544+3_544+6delAAGT variant, which was not detected in the sample of his father and mother. The red box represented the mutant base, and the green box represented the normal base (a).

This patient experienced rapid disease onset during adolescence accompanied by ocular and renal complications, raising strong suspicion of monogenic diabetes. The results showed that the mutation of *HNF1B* (NM_000458.4): c.544+3_544+6delAAGT (Chr17: 37739434-37739437) was detected in the patient, while the parents do not carry this mutation, which is a *de novo* mutation in the proband (**Figure 1**).

According to ACMG guidelines, this variant was classified as follows: PS2 (*de novo* occurrence) and PM2_Supporting (absent from population databases). Based on these criteria, the variant was assessed as a variant of uncertain significance (VUS) (PS2 + PM2_Supporting). Given that the patient’s clinical presentation is relatively classic, yet the ACMG classification currently cannot definitively establish this variant as pathogenic (P) or likely pathogenic (LP). We queried the ClinVar database to further elucidate the relationship between this specific mutation and the observed clinical phenotypes, including diabetes, renal cysts, and hyperuricemia. And the further experiments were needed.

### 3.3 *In vitro* splicing assays

**Figure 2:**
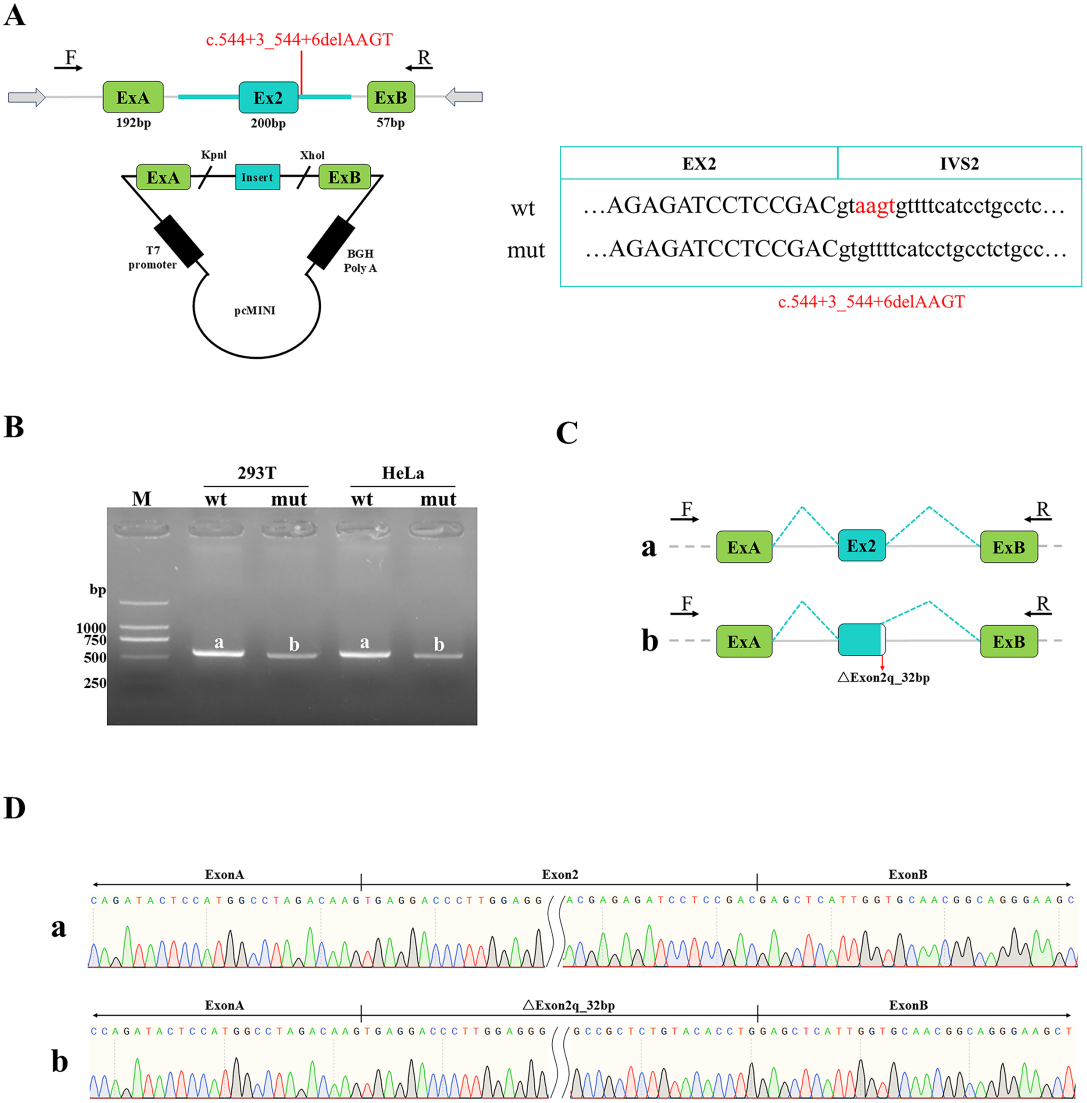
Detection results of pcMINI vector: **(A)** The vector and plasmid construction model is shown, and the top and bottom panels represent the wild-type (wt) and mutant (mut) sequences, respectively, with the red highlighting the difference. T7 denotes the promoter. **(B)** RT-PCR transcript analysis via agarose gel electrophoresis showed that the wild-type (wt) samples in HeLa and 293T cells yielded a single band of the expected size (550 bp), designated as band a. The mutant variant also produced a single band, designated as band b. **(C, D)** Chematic diagrams of splicing patterns corresponding to the sequencing results. Sequencing confirmed that the wild-type band a represents normal splicing: Exon A (192 bp derived from vector) - Exon 2 (200 bp derived from insert) - Exon B (57 bp derived from vector). The mutant band b represents an aberrantly spliced transcript with a 32 bp deletion at the 3'end (right side) of Exon 2, showing a pattern of ExonA (192 bp derived from vector) - ΔExon2 (168 bp derived from insert) - ExonB (57 bp derived from vector).

**Figure 3:**
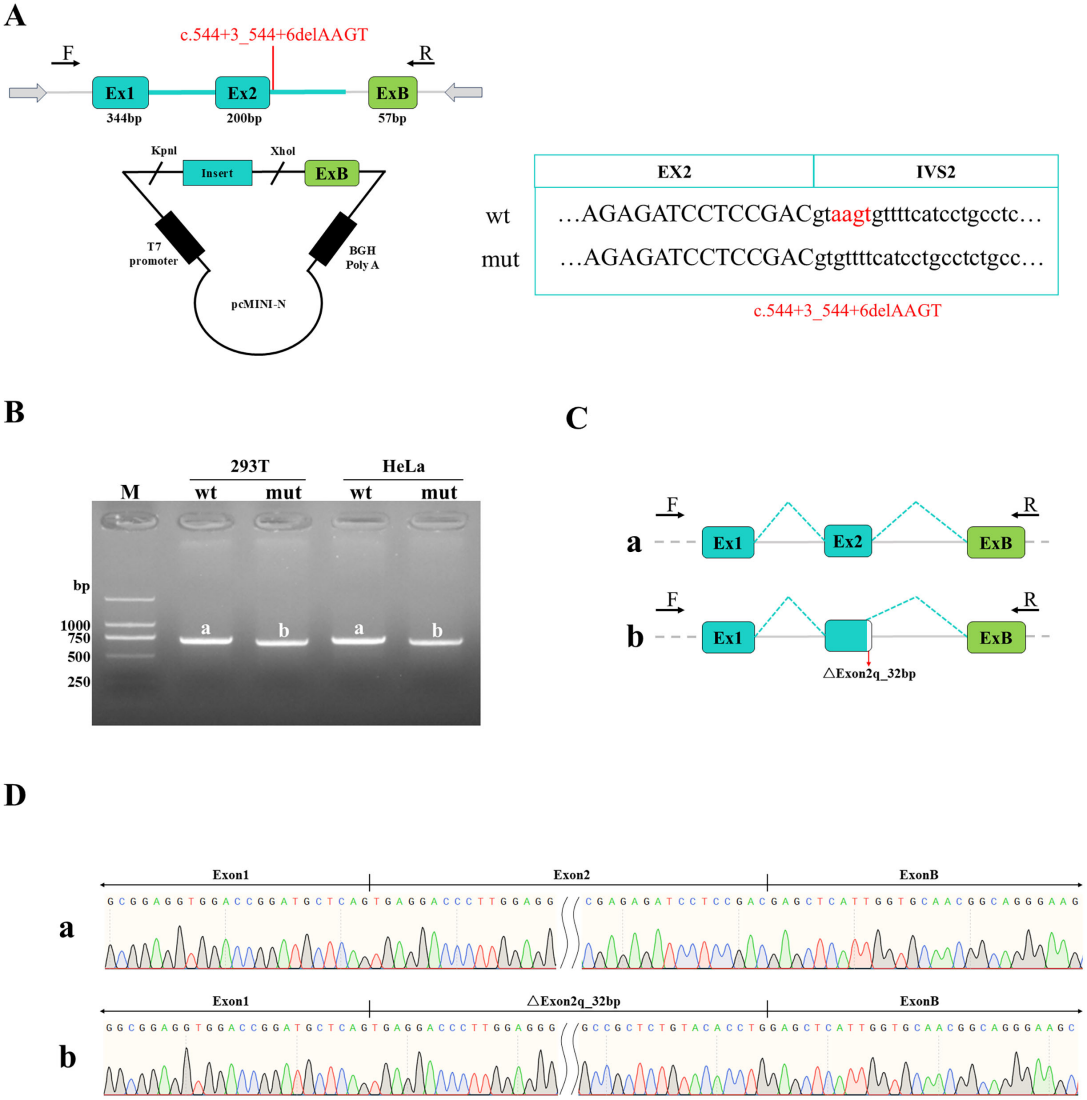
Detection results of pcMINI-N vector: **(A)** The vector and plasmid construction model is shown, and the top and bottom panels represent the wild-type (wt) and mutant (mut) sequences, respectively, with the red highlighting the difference. T7 denotes the promoter. **(B)** RT-PCR transcript analysis via agarose gel electrophoresis showed that the wild-type (wt) samples in HeLa and 293T cells yielded a single band of the expected size (705 bp), designated as band a. The mutant variant also produced a single band, designated as band b. Schematic diagrams of splicing patterns corresponding to the sequencing results. **(C, D)**: Sequencing confirmed that the wild-type band a represents normal splicing: Exon 1 (344 bp derived from vector) - Exon 2 (200 bp derived from insert) - Exon B (57 bp derived from vector). The mutant band b represents an aberrantly spliced transcript with a 32 bp deletion at the 3' end (right side) of Exon 2, showing a pattern of Exon1 (344 bp derived from vector) - ΔExon2 (168 bp derived from insert) - ExonB (57 bp derived from vector).

For the pcMINI construct (**Figure 2**), the wild-type (WT) samples generated a single expected product of 550 bp (designated band a). Sanger sequencing confirmed that band a represented the normal splicing pattern: Exon A (192 bp derived from vector) - Exon 2 (200 bp derived from insert) - Exon B (57 bp derived from vector). In contrast, the mutant samples produced an aberrantly spliced transcript (band b) characterized by a 32-bp deletion at the 3’- end of Exon 2. The resulting modified exon structure for the mutant was: Exon A (192 bp) - ΔExon 2 (168 bp derived from insert) - Exon B (57 bp). Consistent results were observed using the pcMINI-N construct (**Figure 3**). The WT samples produced the expected 705 bp transcript (band a), which corresponded to the normal splicing pattern: Exon 1 (344 bp derived from vector) - Exon 2 (200 bp derived from insert) - Exon B (57 bp derived from vector). Similarly, the mutant samples yielded an aberrantly spliced transcript (band b) harboring the exact same 32-bp deletion at the 3’-end of Exon 2. This resulted in the modified exon structure: Exon 1 (344 bp) - ΔExon 2 (168 bp derived from insert) - Exon B (57 bp).

The minigene assay demonstrated that the c.544+3_544+6delAAGT mutation disrupts normal mRNA splicing of the gene, with consistent results observed from both the pcMINI and pcMINI-N vector systems. This mutation causes a 32-bp deletion at the 3’ end of Exon 2. At the cDNA and protein levels, this alteration is designated as c.513_544del p.Trp171Ter. The 32 bp deletion in Exon 2 induces a frameshift, generating a premature termination codon (PTC) at the end of Exon 2, which is predicted to produce a truncated protein consisting of 170 amino acids.

According to the ACMG guidelines, minigene experimental results demonstrated its impact on splicing (PVS1). In accordance with the ACMG variant classification criteria, this variant is classified as Pathogenic (P) (PS2+PM2_Supporting+PVS1).

### 3.4 Diagnosis

Based on the patient’s primary clinical manifestations and genetic testing results, the diagnosis was established as MODY5 caused by the *HNF1B* c.544+3_544+6delAAGT mutation, accompanied by renal cysts, diabetic nephropathy, status post-cataract surgery, and hyperuricemia. Furthermore, the patient tested positive for insulin autoantibodies (IAA), suggesting the potential coexistence of type 1 diabetes (T1DM). Therefore, close monitoring of T1DM-related markers during follow-up is recommended to guide timely and appropriate therapeutic interventions.

### 3.5 Treatment and follow-up

The patient is currently on a regimen of recombinant human insulin (12 units before dinner and 12 units before meals) along with detemir insulin (22–24 units at bedtime) for glycemic control. Renal cysts require regular follow-up and appropriate medication to slow the progression of renal dysfunction. A kidney transplant may be considered if necessary in the future. Dietary modifications have been implemented and medications such as febuxostat and colchicine have been prescribed to reduce uric acid levels. Given the genetic nature of the condition, genetic counseling has been provided. *HNF1B*-related disorders often present with severe phenotypes and comprehensive treatment remains challenging. The inheritance pattern is autosomal dominant. If the proband has children with a partner who does not carry the mutation, each offspring will have a 50% chance of inheriting the variant. Options such as prenatal diagnosis during pregnancy or assisted reproductive technologies can be considered to prevent transmission. Although genetic testing of the proband’s parents did not detect the same variant in their peripheral blood, the possibility of germline or somatic mosaicism cannot be excluded. Therefore, prenatal diagnosis is recommended in future pregnancies. Following treatment, the patient’s blood glucose is now well-controlled, uric acid levels have decreased, and renal function has improved. However, long-term follow-up has not yet been conducted.

### 3.6 Reports on previous mutations at the same site

Including the present case, a total of seven patients harboring the *HNF1B* (NM_000458.4): c.544+3_544+6delAAGT (Chr17: 37739434-37739437) mutation were identified across databases, including ClinVar and PubMed in **Table 1**. Three patients carried the *HNF1B* c.544 + 3_544 + 6delAAGT mutation: one 6-year-old presented with renal cysts and vesicoureteral reflux but had a normal glomerular filtration rate; the patient’s 30-year-old mother had unilateral renal cysts and gestational diabetes with normal renal function, and her daughter was found to have a unilateral kidney. A 65-year-old patient exhibited a unilateral kidney, gout chronic, renal failure, hyperuricemia, elevated liver enzymes, and hypomagnesemia; the patient’s mother and cousin also had renal failure, and his daughter had a unilateral kidney. Another 33-year-old patient presented with diabetes, renal cysts, chronic renal failure, hyperuricemia, elevated liver enzymes, and hypomagnesemia; his father had end-stage renal failure. Additionally, follow-up of this cohort revealed that patients with renal failure caused by truncated protein-induced premature translation termination had a higher prevalence than those with complete *HNF1B* gene deletions (4). In another study of 114 families with chronic kidney disease, one family carried the same mutation: a 40-year-old female presented with diabetes, annular pancreas, and renal cysts, and a 42-year-old male had diabetes and chronic kidney disease (5). A study screening adolescents and children with diabetes (n = 995) identified five patients with *HNF1B* mutations, one of whom developed cataracts at age 16 and another who had iris defects and vision loss from birth (6). In a separate family with an *HNF1B* mutation, a 13-year-old boy was the proband, presenting with diabetes, renal dysplasia, and cataracts. His uncle had vision impairment due to congenital cataracts, and his grandmother had bilateral cataracts since childhood. The ocular abnormalities were attributed to a heterozygous interstitial microdeletion in the 17q12 region of chromosome 17, which resulted in haploinsufficiency of *HNF1B* (7). The diabetes phenotype associated with *HNF1B* c.544+3_544+6delAAGT mutations is relatively common, while renal cysts and hyperuricemia are less frequent; however, all cases are accompanied by structural or functional renal abnormalities constituting a distinct phenotypic profile. Clinicians can use these renal manifestations to aid diagnosis. Furthermore, this mutation is associated with a high risk of renal failure, underscoring the need for regular follow-up in affected patients to prevent serious adverse events.

**Table 1:** Previous reports of *HNF1B* mutations.

Serial number	Age/Years old	Diabetes	Renal cyst	Renal failure	Hyperuricemia	Cataract	Others	Family history	Ref
1	19	✓	✓	–	✓	✓	–	The grandfather and uncle both have T2DM.	(4)
2	6	–	✓	–	–	–	Vesicoureteral reflux	The mother has unilateral renal cysts and gestational diabetes.	(3)
3	65	–		✓	✓	–	Single kidney	The mother and the cousin have kidney failure, while the daughter has a single kidney.	(3)
4	33	✓	✓	✓	✓	–	Increased liver enzymes and hypoma- gnesemia	The father has end-stage renal failure.	(3)
5	40	✓	✓	–	–	–	Annular pancreas	Chronic kidney disease family	(5)
6	42	✓	–	–	–	–	Chronic nephrosis	Chronic kidney disease family	(5)
7	19	✓	✓	–	✓	✓	–	The maternal grandfather and paternal uncle both have T2DM.	This article

√, yes; -, no; T2DM, type 2 diabetes.

## 4 Discussion

MODY5 caused by mutations in the *HNF1B* gene is inherited in an autosomal dominant manner. Its clinical manifestations are multisystemic and include early-onset diabetes, renal abnormalities (such as renal cysts, renal dysplasia, solitary kidney, horseshoe kidney, and hydronephrosis), abnormal liver function, pancreatic dysplasia, and reproductive tract malformations, among other features (8).

*HNF1B* also known as transcription factor-2 (TCF2), is located on chromosome 17 at region 17q12. The TCF2 protein regulates tissue-specific gene expression in epithelial cells across multiple organs and plays a critical role in early embryonic development (9). During pancreatic development, *HNF1B* promotes the differentiation of human pluripotent stem cells into pancreatic progenitor cells via foregut patterning. *HNF1B* also regulates the expression of glucose transporter 2 (GLUT2) and, in synergy with *HNF1A*, activates GLUT2 transcription (10). Consequently, mutations in *HNF1B* can lead to hyperglycemia. Furthermore, hyperglycemia-induced oxidative stress can activate signaling pathways such as MAPK/ERK. Through mechanisms including mitochondrial superoxide overproduction, activation of the polyol pathway, accumulation of advanced glycation end products (AGEs), abnormal Protein Kinase C signaling, and altered mitochondrial dynamics, this oxidative stress promotes diabetic complications such as cataracts. These pathways further damage lens tissue and inhibit insulin secretion from pancreatic β cells, creating a vicious cycle of metabolic dysfunction. Aberrant *HNF1B* activity concurrently affects both the exocrine and endocrine compartments of the pancreas. Most *HNF1B*-associated pancreatic exocrine dysfunction can be evaluated by identifying a deficiency in fecal elastase (11). Therefore, follow-up assessment of fecal elastase levels in this patient may help evaluate pancreatic development status. Additionally, defective *HNF1B* expression impairs cell proliferation and disrupts early pancreatic developmental programs by altering non-canonical Wnt and Hippo signaling pathways (12). Functional studies targeting these factors may uncover novel therapeutic targets for *HNF1B*-associated diabetes.

The ureteric bud develops into the ureter, renal pelvis, and collecting ducts. Mesenchymal cells proximal to the tips of the ureteric bud cluster to form pretubular aggregates, which sequentially differentiate into comma-shaped and S-shaped bodies, ultimately maturing into Bowman’s capsules and renal tubules. The absence of *HNF1B* expression in the ureteric bud can lead to aberrant branching and impair the conversion of surrounding mesenchymal cells into epithelial cells (mesenchymal-to-epithelial transition) (13). In a French study on genetic testing for kidney diseases (n = 377), 75 patients were found to carry HNF1B mutations. Of these, 75, 37.30% (28/75) had renal failure and 16.00% (12/75) had hyperuricemia (5). This pathogenic link is further supported by zebrafish models with *HNF1B* mutations, which exhibit a renal cyst phenotype (14). Furthermore, *in situ* hybridization of human embryonic tissues reveals high *HNF1B* mRNA expression in collecting ducts compared to lower levels in mesenchymal components (15), further confirming the gene’s pivotal role in early urinary tract development. Interestingly, although *HNF1B* is associated with cystogenesis, knockout of the gene during late-stage renal development does not induce cyst formation, explaining why approximately half of adult patients do not manifest renal cysts. Clinically, patients typically present with renal dysfunction around the age of 40. Approximately 3% to 50% progress to chronic kidney disease (CKD), with some eventually requiring renal transplantation. Among *HNF1B* mutation carriers, the median age of diabetes diagnosis is 24 years, and renal anomalies often precede the onset of diabetic symptoms (16). Research confirms that *HNF1B* mutations elevate intracellular oxidative stress levels, promoting ferroptosis-associated renal cyst formation and fibrosis; notably, *in vitro* experiments demonstrate that ferroptosis inhibitors can mitigate this process (17).

The presence of positive autoantibodies does not preclude a diagnosis of MODY, as single or low-titer antibodies exhibit low specificity for type 1 diabetes (18). A Polish study involving 50 patients with *HNF1B* mutations identified two individuals with autoantibodies (ICA/GAD and ICA/GAD/IAA, respectively) and low C-peptide levels (19). These findings parallel the laboratory results of our patient, suggesting a potential association between *HNF1B* mutations and autoimmunity. Although the prevalence of autoantibodies in MODY patients is generally less than 1% and typically manifests as low-titer single antibodies (18), these cases offer novel insights into the potential coexistence of MODY and T1DM. However, the IAA positivity was detected after the patient had been receiving insulin treatment for nine years. Due to the absence of autoantibody data at the onset of the disease, it remains unclear whether the IAA was induced by prolonged exogenous insulin exposure. However, the potential coexistence of monogenic and autoimmune diabetes still exists.

*HNF1B*-related diseases are also associated with Familial Juvenile Hyperuricemic Nephropathy. Abnormal urate transport may explain the early-onset gout and hyperuricemia that appear disproportionate to the degree of renal dysfunction in patients with *HNF1B* mutations. The *UMOD* encodes uromodulin, which plays a role in renal urate transport. In *HNF1B* knockout mouse models, *UMOD* expression is downregulated, indicating that *HNF1B* transcriptionally regulates *UMOD* (20). Patients with childhood-onset disease often exhibit a rapid decline in renal function. In this study, the patient presented at age 10 and subsequently developed progressive renal impairment, a clinical course consistent with this characteristic profile.

The finding that this novel *HNF1B* splicing variant leads to MODY aligns with established knowledge, as splicing variants are a well-known major cause of MODY. This is supported by a substantial body of prior case reports illustrating similar pathogenic mechanisms. Through a comprehensive search of ClinVar, we identified 37 splicing variants in the *HNF1B* gene. There are three exon mutations, 22 classic splicing mutations, and 11 cases involving mutations in introns or non-classical splicing sites. These findings indicate that while canonical splice site mutations predominate, non-canonical and intronic variants represent a non-negligible cause of disease, warranting increased attention in genetic screening and analysis.

Patients with MODY5 often require insulin therapy at an early stage, typically at relatively low doses. However, if the duration of diabetes is short, oral hypoglycemic agents such as biguanides combined with sulfonylureas may also effectively maintain blood glucose within the normal range. Nonetheless, long-term insulin therapy is generally necessary (1). Good glycemic control helps reduce retinal damage and delays the impairment of the cardiovascular and nervous systems caused by hyperglycemia. However, it does not slow the progression of renal cysts or other structural kidney abnormalities. In cases with poor renal prognosis, renal protective medications are indicated to delay the deterioration of renal function. Some patients with advanced end-stage renal disease may eventually require kidney transplantation (16). For hyperuricemia, medications such as allopurinol and febuxostat can be administered. Additionally improving overall renal function may help reduce the incidence of hyperuricemia. In patients with cataracts strict glycemic control is essential to delay the development of diabetic retinopathy (13). Laser therapy may be necessary in some cases. Recently novel therapeutic agents such as glucagon-like peptide-1 receptor agonists (GLP-1 RAs) or dual glucose-dependent insulinotropic polypeptide (GIP)/GLP-1 RAs have been shown to reduce HbA1c levels in patients with *HNF1B*-related MODY representing a promising direction for future treatment (21).

## 5 Conclusion

In this article, we report a 19-year-old male patient whose clinical phenotype of diabetes, renal cysts, hyperuricemia, and cataracts was closely associated with a *de novo HNF1B* mutation (c.544+3_544+6delAAGT). Subsequent *in vitro* functional assays demonstrated that this deletion variant leads to a frameshift and the production of a truncated protein. While current clinical guidelines have already highlighted the necessity of *HNF1B* screening in patients with early-onset diabetes accompanied by extra-renal features, our research offers new insights into the pathogenicity of non-canonical splice site variants. Additionally, by systematically evaluating the clinical characteristics of this patient and reviewing analogous cases, we have further clarified the robust clinical correlation between this specific mutation and a distinct phenotypic spectrum, namely diabetes, structural renal anomalies (renal cysts), severe hyperuricemia, and early-onset cataracts.

## Data Availability

The datasets presented in this study can be found in online repositories. The names of the repository/repositories and accession number(s) can be found in the article/supplementary material.

## References

[1] Aarthy R Aston-Mourney K Mikocka-Walus A Radha V Amutha A Anjana RM . Clinical features complications and treatment of rarer forms of maturity-onset diabetes of the young (MODY) - A review. J Diabetes Complications. (2021) 35:107640. doi: 10.1016/j.jdiacomp.2020.107640, PMID: 32763092

[2] Laver T Wakeling M Knox O De-Franco E Flanagan S Colclough K . Redefining the pathogenicity of Maturity Onset Diabetes of the Young (MODY) genes: BLK, PAX4 and KLF11 do not cause MODY. Diabetic Med. (A15(P112)). doi: 10.1111/dme.2_13570

[3] Sharma M Maurya K Nautiyal A Chitme HR . Monogenic diabetes: A comprehensive overview and therapeutic management of subtypes of mody. Endocr Res. (2025) 50:1–11. doi: 10.1080/07435800.2024.2388606, PMID: 39106207

[4] Heidet L Decramer S Pawtowski A Morinière V Bandin F Knebelmann B . Spectrum of HNF1B mutations in a large cohort of patients who harbor renal diseases. Clin J Am Soc Nephrol. (2010) 5:1079–90. doi: 10.2215/CJN.06810909, PMID: 20378641 PMC2879303

[5] Connaughton DM Kennedy C Shril S Mann N Murray SL Williams PA . Monogenic causes of chronic kidney disease in adults. Kidney Int. (2019) 95:914–28. doi: 10.1016/j.kint.2018.10.031, PMID: 30773290 PMC6431580

[6] Raile K Klopocki E Holder M Wessel T Galler A Deiss D . Expanded clinical spectrum in hepatocyte nuclear factor 1b-maturity-onset diabetes of the young. J Clin Endocrinol Metab. (2009) 94:2658–64. doi: 10.1210/jc.2008-2189, PMID: 19417042

[7] Hogendorf A Kosińska-Urbańska M Borowiec M Antosik K Wyka K Młynarski W . Atypical phenotypic features among carriers of a novel Q248X nonsense mutation in the HNF1B gene. Endokrynol Pol. (2015) 66:15–21. doi: 10.5603/EP.2015.0004, PMID: 25754277

[8] Bonnefond A Unnikrishnan R Doria A Vaxillaire M Kulkarni RN Mohan V . Monogenic diabetes. Nat Rev Dis Primers. (2023) 9:12. doi: 10.1038/s41572-023-00421-w, PMID: 36894549

[9] Izzi C Dordoni C Econimo L Delbarba E Grati FR Martin E . Variable expressivity of HNF1B nephropathy from renal cysts and diabetes to medullary sponge kidney through tubulo-interstitial kidney disease. Kidney Int Rep. (2020) 5:2341–50. doi: 10.1016/j.ekir.2020.09.042, PMID: 33305128 PMC7710890

[10] Ono Y Kataoka K . MafA, NeuroD1 and HNF1β synergistically activate the Slc2a2 (Glut2) gene in β-cells. J Mol Endocrinol. (2021) 67:71–82. doi: 10.1530/JME-20-0339, PMID: 34223824

[11] Zeinali Nia E Najjar Sadeghi R Ebadi M Faghihi M . ERK1/2 gene expression and hypomethylation of Alu and LINE1 elements in patients with type 2 diabetes with and without cataract: Impact of hyperglycemia-induced oxidative stress. J Diabetes Investig. (2025) 16:689–706. doi: 10.1111/jdi.14405, PMID: 39804191 PMC11970314

[12] El-Khairi R Olszanowski E Muraro D Madrigal P Tilgner K Chhatriwala M . Modeling HNF1B-associated monogenic diabetes using human iPSCs reveals an early stage impairment of the pancreatic developmental program. Stem Cell Rep. (2021) 16:2289–304. doi: 10.1016/j.stemcr.2021.07.018, PMID: 34450036 PMC8452540

[13] Bingham C Ellard S van’t Hoff WG Simmonds HA Marinaki AM Badman MK . Atypical familial juvenile hyperuricemic nephropathy associated with a hepatocyte nuclear factor-1beta gene mutation. Kidney Int. (2003) 63:1645–51. doi: 10.1046/j.1523-1755.2003.00903.x, PMID: 12675839

[14] Clissold RL Hamilton AJ Hattersley AT Ellard S Bingham C . HNF1B-associated renal and extra-renal disease-an expanding clinical spectrum. Nat Rev Nephrol. (2015) 11:102–12. doi: 10.1038/nrneph.2014.232, PMID: 25536396

[15] Dressler GR . The cellular basis of kidney development. Annu Rev Cell Dev Biol. (2006) 22:509–29. doi: 10.1146/annurev.cellbio.22.010305.104340, PMID: 16822174

[16] Poitou C Francois H Bellanne-Chantelot C Noel C Jacquet A Clauin S . Maturity onset diabetes of the young: clinical characteristics and outcome after kidney and pancreas transplantation in MODY3 and RCAD patients: a single center experience. Transpl Int. (2012) 25:564–72. doi: 10.1111/j.1432-2277.2012.01458.x, PMID: 22432796

[17] Chen X Yang C Wei Q Huang M Wang A Zhang M . A novel mutation in HNF1B promotes ferroptosis-mediated renal mesangial cells fibrosis. Biochem Biophys Res Commun. (2024) 736:150803. doi: 10.1016/j.bbrc.2024.150803, PMID: 39490151

[18] Sanri A Cetin TK Acikgoz EG Mutlu MB Sezer O . Revealing monogenic diabetes: clinical and genetic features of pediatric MODY cases in Türkiye: single center experience. Pediatr Diabetes. (2025) 2025:4035026. doi: 10.1155/pedi/4035026, PMID: 41367630 PMC12685419

[19] Sztromwasser P Michalak A Małachowska B Młudzik P Antosik K Hogendorf A . A cross-sectional study of patients referred for HNF1B-MODY genetic testing due to cystic kidneys and diabetes. Pediatr Diabetes. (2020) 21:422–30. doi: 10.1111/pedi.12959, PMID: 31825128 PMC7217165

[20] Verhave JC Bech AP Wetzels JF Nijenhuis T . Hepatocyte nuclear factor 1β-associated kidney disease: more than renal cysts and diabetes. J Am Soc Nephrol. (2016) 27:345–53. doi: 10.1681/ASN.2015050544, PMID: 26319241 PMC4731131

[21] Mehdi AZ Deng L Chase CL Foss-Freitas MC Gregg B Naylor RN . GLP-1 RA and dual GIP/GLP-1 RA treatment in MODY: a descriptive case series. BMJ Open Diabetes Res Care. (2025) 13:e004885. doi: 10.1136/bmjdrc-2024-004885, PMID: 40274278 PMC12020758

